# The Effects of Experimental Irrigation on Plant Productivity, Insect Abundance and the Non-Breeding Season Performance of a Migratory Songbird

**DOI:** 10.1371/journal.pone.0055114

**Published:** 2013-01-25

**Authors:** Scott Wilson, Peter P. Marra, T. Scott Sillett

**Affiliations:** Migratory Bird Center, Smithsonian Conservation Biology Institute, National Zoological Park, Washington DC, United States of America; University of Milan, Italy

## Abstract

Migratory bird populations are often limited by food during the non-breeding season. Correlative evidence suggests that food abundance on territories varies among years in relation to rainfall, which affects plant productivity and arthropod biomass. At the Font Hill Nature Preserve in Jamaica, we used an irrigation experiment to test the hypothesis that rainfall affects the condition of wintering American redstarts (*Setophaga ruticilla*) via intermediate effects on plant productivity and arthropod abundance. Experimental plots were irrigated in late February and early March to simulate a mid-season pulse of 200 mm of rain. Irrigation maintained soil moisture levels near saturation and had immediate effects on plant productivity. Cumulative leaf abscission over the dry season was 50% lower on experimental plots resulting in greater canopy cover, and we observed significantly higher ground level shoot growth and the flushing of new leaves on about 58% of logwood (*Haematoxylon campechianum*) individuals. Arthropod biomass was 1.5 times higher on irrigated plots, but there was considerable inter-plot variability within a treatment and a strong seasonal decline in biomass. Consequently, we found no significant effect of irrigation on arthropod abundance or redstart condition. We suspect that the lack of an irrigation effect for taxa higher on the trophic chain was due to the small spatial scale of the treatment relative to the scale at which these taxa operate. Although redstart condition was not affected, we did observe turnover from subordinate to dominant territorial individuals on experimental plots suggesting a perceived difference in habitat quality that influenced individual behavior.

## Introduction

Populations of Nearctic-Neotropical migratory songbirds are influenced by a multitude of factors that operate throughout the annual cycle [1], [2], [3], [4]. A critical limiting factor during the non-breeding season is food abundance and more broadly, habitat quality [5], [6]. In many passerine species, individuals segregate among habitats of varying quality during winter with more dominant individuals excluding subordinates from higher quality sites [7], [8], [9]. Spatial variation in habitat quality affects a number of performance metrics including body condition [3], [10], [11], [12], survival [13], timing of spring migration and reproductive performance on the breeding grounds [1], [14]. The quality of a particular habitat can also vary temporally among years, due in large part to the effects of precipitation on plant productivity and insect abundance. In Jamaica, arthropods can be up to five times more abundant in wet years and this has direct effects on winter body condition and migratory timing of insectivorous bird species such as the American redstart *Setophaga ruticilla* [15], [16]. At broader scales, annual variability in conditions across the Caribbean wintering range translate into effects on population abundance for Neotropical migrants on the breeding grounds [17].

Despite mounting correlative evidence for the role of winter rainfall in determining migratory bird performance, research is needed to clearly elucidate the fundamental mechanisms within the bottom-up transfer from precipitation to birds, especially how rainfall affects plant productivity and how plant productivity in turn influences insect abundance. Many plant species in the seasonally dry forests of the Caribbean and Central America exhibit leaf abscission during the winter dry season. Leaf abscission is thought to be a plant’s response to inadequate water storage capacity to maintain leaves during drought conditions [18], [19], [20], and could also allow for the re-allocation of resources to fruit and seed development [19, [21], [22]. Several proximal cues have been proposed as triggers for leaf abscission, including drought, photoperiod, leaf age and plant water status [23], [24], [25], [26]. Species vary in the timing of leaf flush with some growing new tissue just after the onset of the wet season, typically in early May, while others do so at the end of the dry season when soil moisture levels are at their lowest [26], [27]. Some species also respond to unusual rainfall events and will flush new leaves during the middle of the dry season [20], [28]. Following this, experimental irrigation studies have shown that artificially increasing soil moisture can delay leaf abscission and encourage new leaf growth, but the intensity and timing of the response varies among plant species and habitat types [24], [29], [30], [31].

Most Neotropical migratory passerines are insectivorous and thus, the degree to which plant productivity affects individuals should be determined, in part, by the intermediate arthropod response. Arthropod abundance in the seasonal Neotropics is usually correlated with rainfall and is highest during the early to mid-wet season and lowest during the latter half of the dry season in March and April [32], [33], [34]. Inter-annual variation in arthropod abundance is also common and typically related to the total precipitation among years [15], [35]. To our knowledge, only one study in tropical regions of the western hemisphere experimentally investigated the effects of simulated rainfall on insect abundance and those results showed only a weak response of some taxa to irrigation [36]. Beier et al. [37] emphasize the need for further studies that manipulate precipitation, particularly in tropical and south temperate regions where we have a more limited understanding of the intricate mechanisms by which rainfall affects ecosystem processes (see also [38]).

We used an irrigation experiment to test the hypothesis that seasonal precipitation limits plant productivity, and in turn insect abundance and the condition of migratory songbirds in lowland Jamaican scrub forest during the winter dry season. The dry season in this region extends from January through April and is characterized by the abscission of leaves on many plant species and the cessation of plant growth. Following earlier results showing a positive response of plants and arthropods to pulses of dry season rainfall [15], we predicted that irrigation over a 2-week period (equivalent to 200 mm) would i) reduce leaf abscission, ii) encourage new leaf flushing, iii) increase the abundance of insects in areas with higher plant productivity and iv) lead to greater body condition for a Neotropical migratory songbird. We also measured nitrogen 15 (N-15) abundance across trophic levels in control and irrigated plots. N-15 becomes enriched in land based water sources relative to rainfall [39] and we used this to predict that N-15 should also be higher on experimental plots if the irrigation water had been transferred from the soil up the trophic chain.

## Methods

### Study Site

The study was conducted in 2009 at the Font Hill Nature Preserve (18° 02′N, 77° 57′W), 13 km west of Black River, St. Elizabeth Parish, Jamaica. The southwest coast is one of the driest regions of Jamaica with annual precipitation <1000 mm. Precipitation is seasonal and monthly rainfall exceeds 100 mm during the August to November wet season and a shorter wet season in May. Monthly rainfall is typically less than 25 mm during the dry season from December to mid-April (Jamaican Meteorological Service). Temperatures are relatively constant throughout the year with daily averages ranging between 25 and 29 degrees Celsius.

Our study was part of a larger, long-term study (1987-present) on the wintering ecology of American redstarts [1], [10]. Primary habitats at the study site consist of black mangrove (*Avicennia germinans*) forest and second growth scrub, but the irrigation experiment was situated only in second growth scrub habitat containing trees ranging from 2 to 5 cm dbh and 2 to 8 m in height that formed a dense understory layer of vegetation. Cattle grazing and cutting for fence posts and firewood also occurred at the site, creating smaller grassy openings amidst larger patches of scrub. Vegetation in second growth scrub habitat was dominated by logwood (*Haematoxylon campechianum*), a species native to dry forests of Mexico and Central America that was introduced to Jamaica in the 19^th^ century [40]. Other less common tree species include *Bursera simaruba*, *Terminalia latifolia* and *Crescentia alata*. For additional detail on the study area see [10].

### Avian Study Species

American redstarts breed in deciduous and mixed forests across eastern, boreal and western mountainous regions of North America. Their winter range includes the Caribbean, Mexico, Central America and northern South America, but winter abundance is highest in the Greater Antilles and in western Mexico [41]. Most individuals will arrive on the wintering grounds in late September and October, and depart on spring migration in March and April towards the end of the dry season [41]. Redstarts overwinter in a range of wooded habitats in Jamaica, but abundance is highest in black mangrove and coastal scrub forest [13], the two habitat types in our study area. Individuals are territorial in winter and display habitat segregation by sex and age. Older, after second year males typically select higher quality territories in black mangrove forest, while younger males and females are more common in lower quality scrub forest [7], [9], [10].

### Experimental Design

We conducted the irrigation experiment from mid-January through mid-May. Our objective was to assess the effects of supplemented water on plant growth, insect biomass and bird performance during the peak of the dry season in late February through early April. We simulated 200 mm of rainfall across ten 25 m-diameter plots at two sites separated by approximately 300 m. Dry season precipitation in the 20–50 mm range is the expected minimum amount required for new leaf flushing of drought-deciduous trees [28], [31], [42]. Within each site, the experimental plots were at least 50 m apart and spread over 1.5 to 2 hectares. We also established ten 25 m diameter control plots in second growth scrub over a 2.5 hectare area. Control and experimental plots were in the same stand of logwood forest covering approximately 6 hectares and, forest age and composition were similar for both.

Irrigation water was drawn from two freshwater ponds (one at each site) estimated to contain 1.2 and 0.6 million liters respectively in mid-February. For irrigation we used a 6.5 hp Pacer gas pump (Pacer Pumps, Lancaster, Pennsylvania, USA) to draw water at a maximum rate of 575 liters per minute and a pressure of 40 P.S.I. Water was pumped along a 50 mm-diameter main line pipe with 20 mm branch lines diverting water to an elevated (1 m) impact sprinkler. The sprinkler was continually rotating and we avoided placing it immediately next to thick vegetation to ensure that it distributed water evenly across the plot. On irrigation days, watering started at 6am and continued for 3 to 4 hours. The timing at the two sites was staggered such that each received irrigation during two pulses of 9 days with approximately 10 days without irrigation in-between. At experimental site 1, the two irrigation periods were 19 February to 27 February and 11 March to 19 March. At experimental site 2, the periods were 1 March to 9 March and 20 March to 28 March. A tensiometer (Soil Moisture Equipment Corporation, Model 2725ARL) was placed in one experimental and one control plot at a depth of approximately 15 cm to examine soil moisture conditions throughout the study. On all plots, we sampled vegetation, arthropods and birds before and after the irrigation periods.

### Vegetation Sampling

We measured leaf fall every 7 days from 27 January to 27 April on nine irrigation plots and eight control plots. Leaves were collected using leaf traps placed near the center of each plot. Traps were constructed of wire mesh (∼2.5 mm mesh) and were 0.5 m^2^ in size with a 10 cm side. After leaves were collected from the traps, they were left to dry for 1–2 weeks at approximately 30–40°C. Once dry, leaves of all species combined were weighed to 0.1 g. Comparisons of leaf fall between control and experimental plots were based on the cumulative leaf fall over the study and the mean weakly leaf fall after the onset of irrigation at both sites.

As estimates of vegetation productivity, we measured canopy cover, new growth of logwood leaves, ground layer growth and the extent of understory and subcanopy vegetation. Hemispherical canopy cover photos were taken from the center of each plot using a digital camera with a fish-eye lens and placed on a tripod ∼0.75 m off the ground. A single photo was taken on each plot prior to irrigation and again 10 days after irrigation ended. The photos were analyzed using the Gap Light Analyzer program version 2.0 to estimate canopy cover and leaf area index [43]. Ground layer growth was measured at four points in the plot. A 1 m-diameter circle was defined 8 m out from the center of the plot in each of the cardinal directions. Within this circle, we measured the number of independent shoots that contained green vegetation at <30 cm height. This height was selected to avoid including larger tree and shrub species, which were measured using the methods previously described. For each plot, we averaged the number of shoots across the four circles to derive a single estimate that was used for analyses. In the same four locations, we measured sub-canopy and shrub layer vegetation using a Robel pole [44] that was 3 m in height with six 0.5 m sections. The pole was placed vertically at the center of each of the four points and we recorded whether green vegetation touched the pole in each of the six sections. We then summed the total across the four points for each plot and used this value for analyses. Thus, Robel pole measures could vary between 0 and 24 with higher values indicating denser levels of green understory vegetation. We also measured whether there was new leaf growth after irrigation by randomly selecting 10 individual logwood trees and examining them for any evidence of new growth and the percent of leaves that represented new growth. Measurements of canopy cover, ground layer shoot growth and Robel measures were recorded prior to irrigation, within two weeks post-irrigation and after the onset of the wet season in early May. Our estimates of new growth were only conducted in the latter two periods as there was no fresh growth on any plot prior to irrigation.

### Arthropod Abundance

For the second component of our study, we examined whether irrigation affected the abundance of subcanopy and canopy arthropods. Surveys were conducted early in the dry season (7–9 February) before the start of irrigation and during the late dry season after irrigation (5–6 April). Arthropod sampling methods followed a consistent protocol used previously at this site and are intended to survey smaller arthropods that are regularly selected by redstarts at middle, subcanopy and canopy levels in the vegetation [15], [41]. Two observers made 20 passes of a sweep net within the 12 m-radius plot, The sweep net was attached to a 3 m pole, allowing the samples to be collected from canopy and sub-canopy layers. The volume of the vegetation surveyed is a small fraction of the volume on the plot and territory and should have a minimal influence on arthropod abundance after sampling. After collection, the contents of the sweep net were overturned into a plastic bag and placed in a freezer (−10°C) for at least 48 hours. After freezing, we removed all vegetation in the sample, placed the remaining arthropods in 70% ethanol and later dried the sample at 60°C prior to weighing and sorting the individuals. The sample was then weighed for a total biomass and sorted into groups representing orders and sub-orders that were identifiable and regularly recorded in samples. The vast majority of arthropods weighed less than 5 mg but in 3 of the 20 plots (2 control and 1 irrigation plot), a single large individual (either Lepidopteran or Orthopteran) was present in the sample and for these three samples, represented 76, 95 and 96 percent of the total biomass. The presence of these individuals skewed the total biomass and made statistical comparisons difficult. Therefore, we include measures of biomass with and without these individuals but only ran statistical analyses with them excluded.

### Avian Response

All experimental and control plots were centered on the winter territory of an American redstart and our aim was to test whether individuals on irrigation plots maintained or improved body condition after irrigation relative to those on control plots. Because this study was part of a larger project, many individuals were already color-marked with a numbered United States Geological Survey aluminum leg band and two color leg bands. In these cases, we conducted behavioural observations of the individual in January and early February to identify the location of their territory. We also monitored the movements of individuals on territories outside of the long-term study area and these were assigned to experimental irrigation plots. Once the general territory boundaries were identified for all control and experimental plots, we captured each individual using mist-nets accompanied by playbacks and a redstart decoy. After capture, unmarked birds were uniquely marked with aluminum and color leg bands, and all individuals were aged, sexed, measured (wing chord, tail length, tarsus length, bill length, width and depth to ±0.1 mm), weighed to the nearest 0.1 g using an Ohaus digital scale and then released. We conducted behavioral observations on newly marked individuals on both control and experimental sites to ensure that they remained on territory. During the irrigation study, we located all banded, territorial individuals every 2–4 days to determine whether any territory shifts occurred. We attempted to re-capture and re-measure each territorial bird within two to three weeks after irrigation. Weight and morphometric measurements were used to derive an index of condition by taking the residuals from a regression of mass against wing length, tarsus and tail length. Redstarts were also monitored regularly during the experiment to identify any possible impact of disturbance from the sprinklers on behavior but we did not observe any notable effects.

### Isotopic and Statistical Analyses

We used nitrogen isotope comparisons of plants, hemipterans, spiders and birds to examine trophic transfer within experimental and control plots. N-15 becomes more enriched in land-based water sources relative to rainfall [39] and we expected this effect to result in higher N-15 abundance for all trophic levels on the irrigated compared to the control plots. For each sample, approximately 4 mg of plant tissue and 0.5 mg of animal tissue were ground into a fine powder, packaged in tin capsules and weighed to the nearest 1 µg. Samples were run on a Thermo Delta V Advantage mass spectrometer in continuous flow mode coupled to a Costech 4010 Elemental Analyzer (EA) via a Thermo Conflo IV. All isotopic analyses were conducted at the Smithsonian OUSS/MC Stable Isotope Mass Spectrometry Laboratory. Samples are repeatable to ±0.2‰ based on measurements for standards. Values reported in the text and tables are the ratio of N-15 to N-14 expressed as a percentage.

Several comparisons involved the extent of change in the metric before and after irrigation including canopy cover, leaf area index, Robel pole measurements, insect abundance and redstart body condition. In these cases, the difference was compared using a paired t-test. Mean weekly leaf fall, the number of shoots per m^2^ after irrigation and nitrogen isotopes only represented a single comparison between control and irrigated plots and were conducted using a two-sample test. We also used a two-sample t-test to compare canopy cover, leaf area index and Robel pole measures at the start of the wet season between control and treatment plots since this was not intended as a comparison of the change from the earlier period. Statistical analyses were conducted using program R, version 2.11.1.

## Results

### Soil Moisture, Vegetation Response and Isotopic Values

Natural rainfall from January through March totaled 146 mm, while the long-term average between 1994 and 2009 was 166 mm (sd = 93 mm). Thus, our study year could be considered to have had typical rainfall levels during the peak dry season. Each experimental plot received approximately 30,000 L of fresh water during each irrigation period (60,000 L total), which would approximately simulate a 200 mm increase in rainfall. This increase would be equivalent to an extreme wet year, exceeded only slightly by the level in 1995 at 350 mm.

**Figure 1:**
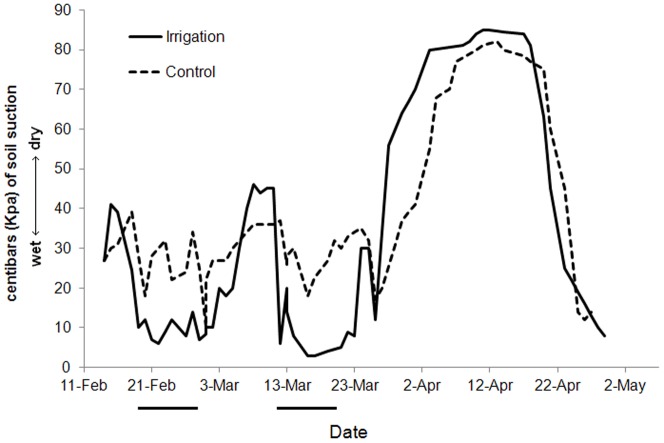
Soil moisture conditions on irrigated and control plots at the Font Hill Nature Preserve, Jamaica. Soil moisture is measured in centibars (Kpa) of soil suction with lower values indicating moister conditions. Irrigation on the plot took place from 19 February to 27 February and 11 March to 19 March (represented by the two black bars under the x-axis).

Irrigation had an immediate effect on soil moisture (Figure 1). Within a few hours after the onset of irrigation, soil moisture values dropped below 15 centibars (Kpa) of soil suction (lower values indicate wetter soil conditions) and remained at 5–10 Kpa throughout the irrigation period. There was a 2 to 3 day lag after irrigation ended before soil moisture levels began to decrease. Values between irrigation periods were temporarily higher on the irrigated plot relative to the control plot until the second round of irrigation began, at which point they dropped back to 5–10 Kpa. Irrigation ceased in mid-March and soil dryness continued to increase on both experimental and control plots until rainy conditions started in late April (Figure 1). Note that variation in the control plot during the irrigation periods was due to small amounts of rainfall.

**Figure 2:**
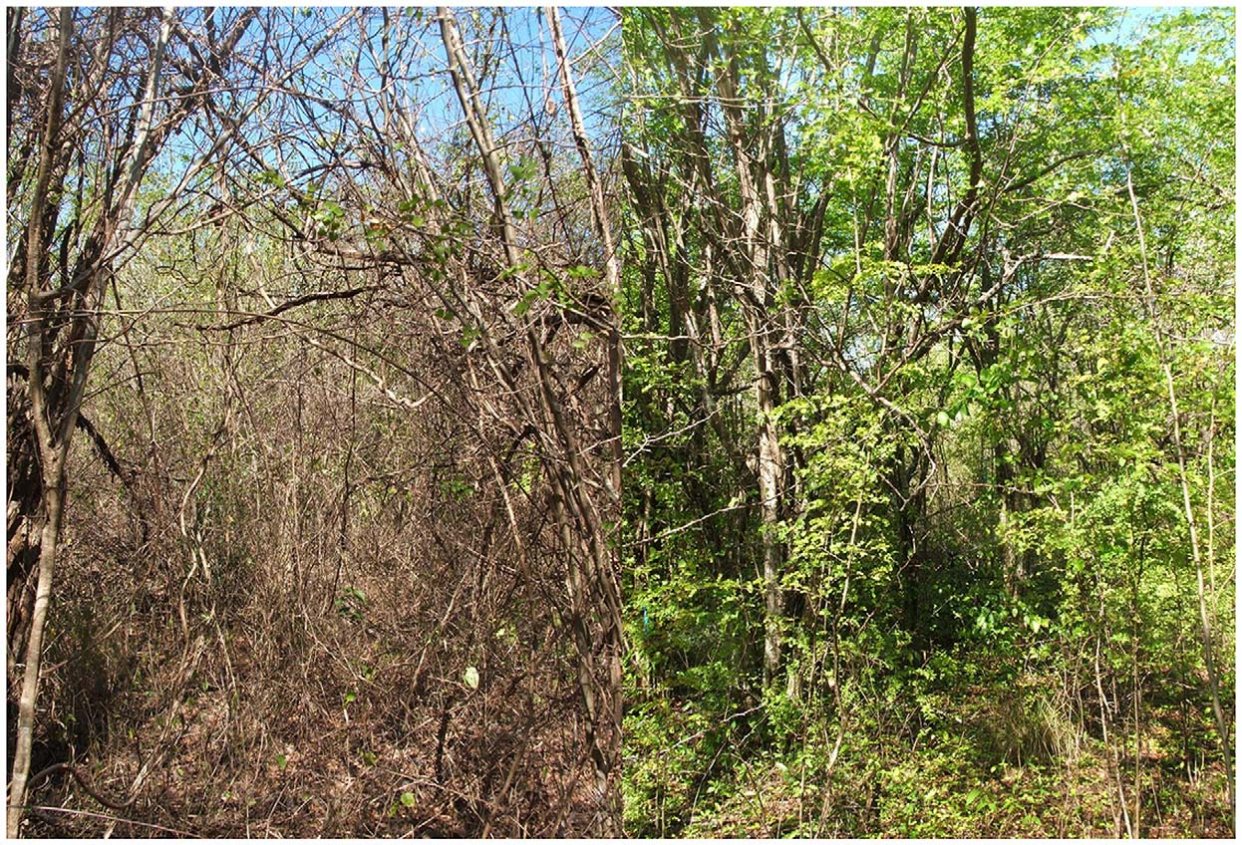
Effects of experimental irrigation on dry season scrub habitat at the Font Hill Nature Preserve, Jamaica. A control plot is shown on the left and an irrigated plot on the right. Photos were taken in late March after the second round of irrigation.

**Figure 3:**
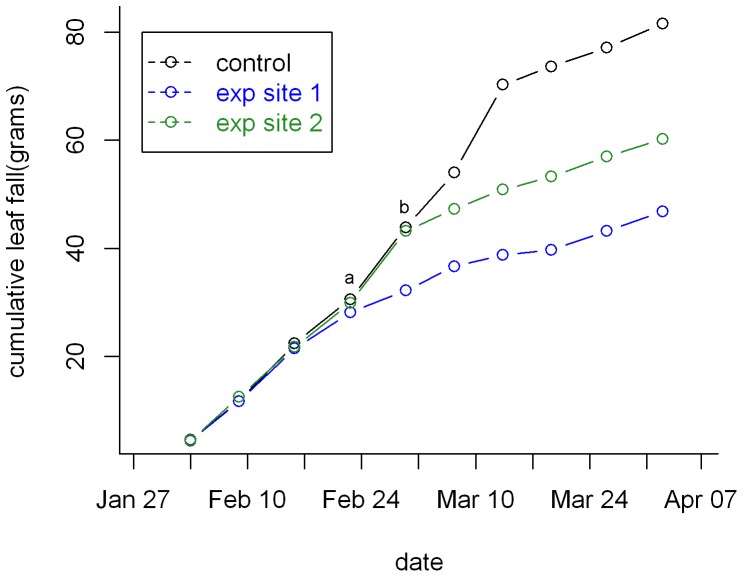
Cumulative leaf fall on control and irrigation plots. Leaves were collected weakly in 0.5 m^2^ traps and values have been scaled to grams/m^2^. a and b represent the onset of irrigation for experimental sites 1 and 2 respectively.

**Figure 4:**
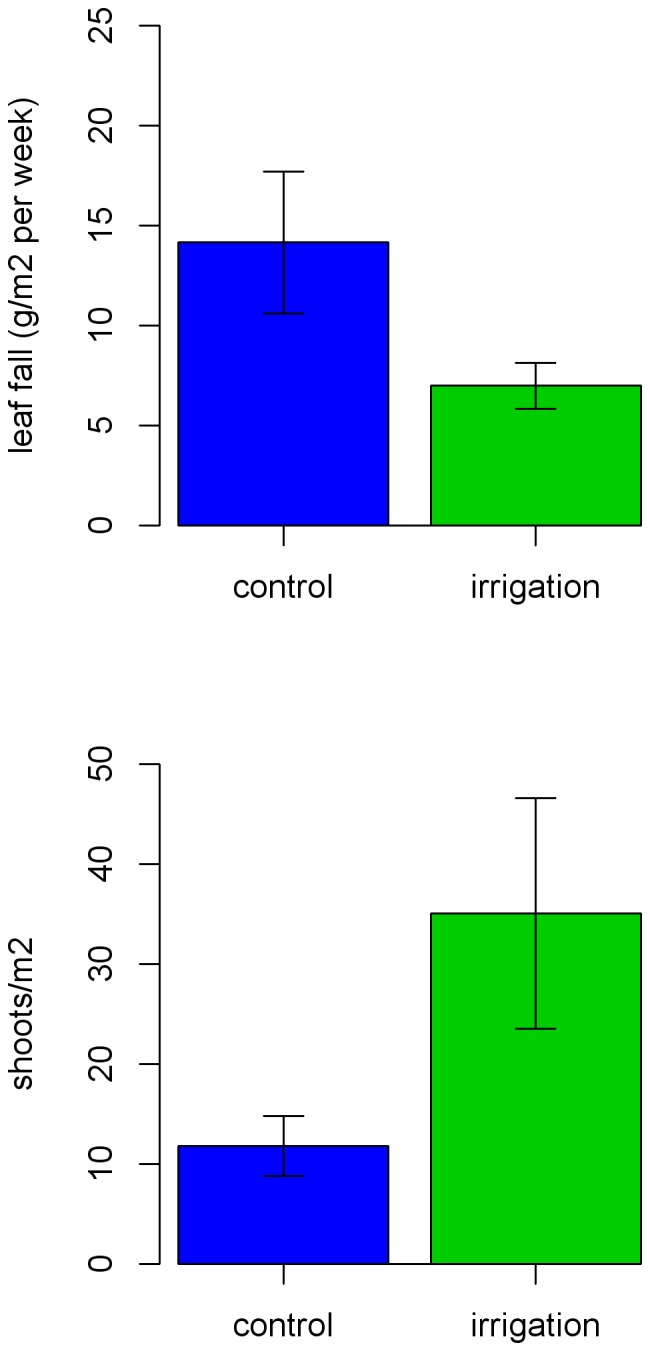
Variation in weekly leaf fall and ground level shoot growth after irrigation on control and treatment plots at Font Hill, Jamaica (values include mean and 95% confidence interval).

**Figure 5:**
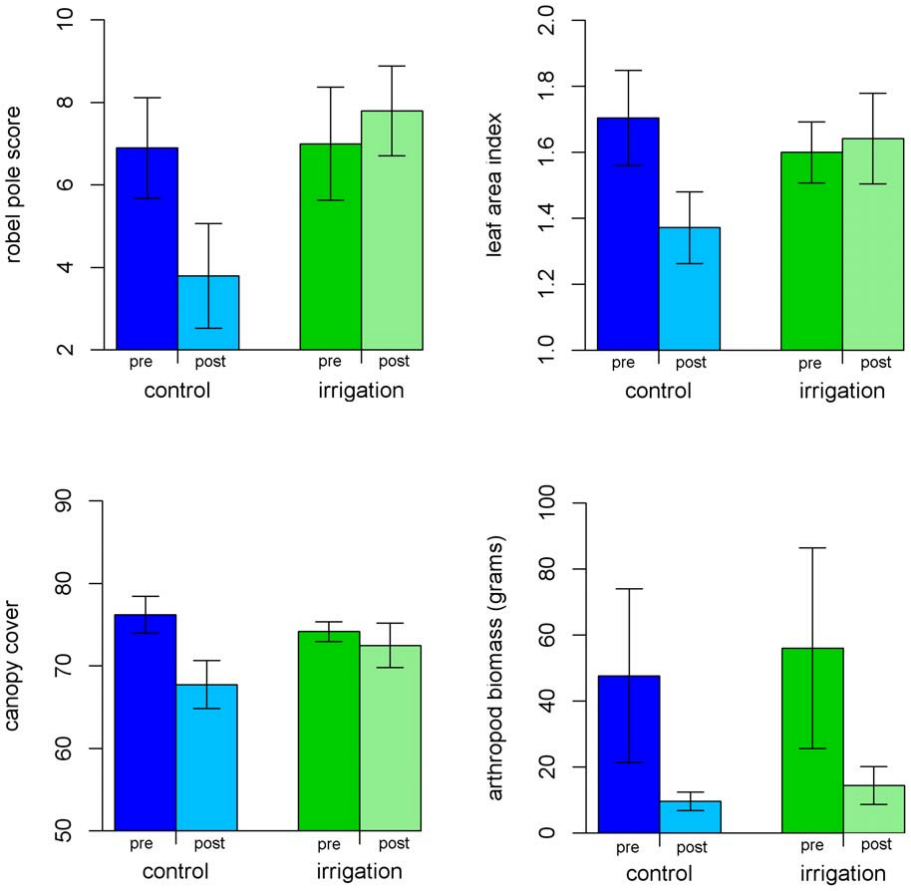
Variation in canopy cover, leaf area index, Robel measures of vegetation greenness and arthropod biomass on control and irrigated plots at Font Hill, Jamaica (values include mean and 95% confidence interval). Columns labelled “pre” are the early season measurements prior to irrigation (late January/early February), while columns labelled “post” are the late season conditions after irrigation (late March/early April).

Irrigation had strong effects on all measures of plant productivity (Figures 2–5). Prior to irrigation, weekly leaf abscission was equal across control and experimental plots. The extent of leaf abscission then declined about one week after the onset of irrigation (Figure 3) and the mean weakly leaf abscission was less than half of what it was on control plots for the entire period between 6 March and 27 April when measurements stopped (Figure 4, two-sample t-test, t = −3.76, p = 0.002, df = 14). Irrigation also had a significant influence on ground level vegetation with over three times the number of shoots per m^2^ compared to control plots (Figure 4, t = −3.82, p = 0.001, df = 18). All measures of vegetation at subcanopy and canopy layers including canopy cover (paired t-test, t = −6.63, p<0.001, df = 9), leaf area index (t = −3.87, p = 0.004, df = 9) and Robel pole scores (t = −2.19, p = 0.056, df = 9) also showed significant differences (marginal for Robel score) between treatments with the extent of green vegetation declining between the pre- and post-irrigation periods on the control plots but remaining relatively constant on irrigated plots. This latter effect was due to lower leaf abscission and some new growth at different layers. (Figures 4,5). Accordingly, 58% of *H. campechianum* individuals had new leaf growth after irrigation on experimental plots, while no individuals did on control plots.

All measurements were repeated between 4 May –9 May, after the rains had returned, and we found no significant differences between control and irrigated plots during this interval for canopy cover (control: 80.56±0.81 (SE), irrigation: 79.25±0.61, t = 1.30, p = 0.22, df = 18), leaf area index (control: 1.96±0.11 (SE), irrigation: 1.91±0.03, t = 0.51, p = 0.63, df = 18), Robel pole measures (control: 6.6±0.5 (SE), irrigation: 7.2±0.6, t = −0.81, p = 0.43, df = 18) or ground level shoots per m^2^ (control: 115.3±16.4 (SE), irrigation: 107.1±17.6, t = 0.34, p = 0.73, df = 18). All sampled individuals of *Haematoxylon campechianum* had recent leaf growth in control plots in early May, while only 69% did on irrigated plots.

Nitrogen isotopes were used to confirm trophic transfer of irrigation water through the use of water sources that are expected to differ in N-15 enrichment (i.e. higher for land based water sources used for irrigation). N-15 values were significantly higher on irrigated plots for plants (two-sample t-test, t = 3.63, p = 0.002, df = 24), hemipterans (t = 3.41, p = 0.002, df = 21) and redstarts (t = 2.78, p = 0.010, df = 21), but not for Aranea (t = 1.26, p = 0.22, df = 20), although the mean value was higher on irrigated plots (Table 1). This result provides strong evidence that pond water used for irrigation was incorporated into the tissues of plants and hemipterans that were sampled within the plot as well as for redstarts whose territories were centered on the plots. It is not clear why Aranea showed no significant differences, but it’s possible that they acquired resources over a broader area or that uptake did not occur quickly enough to be reflected in their tissues by the time we collected samples.

**Table 1:** Nitrogen-15 isotope values for four taxonomic groups on control and irrigated plots at Font Hill, Jamaica.

Group	Control	Irrigated
Logwood	1.63±0.53 (13)	3.81±0.40 (13)*
Hemiptera	2.78±0.42 (13)	5.08±0.57 (9)*
Aranea	6.23±0.31 (12)	6.94±0.51 (9)
American redstart	6.69±0.18 (10)	7.26±0.11 (9)*

Values include the mean and standard error with the sample size in brackets. Stars indicate significant differences (p<0.05).

### Arthropod Responses

Canopy arthropod biomass declined strongly through the season dropping by 80% and 74% on control and irrigated plots respectively (Figure 5, Table S1). This decline was primarily related to a change in the abundance of small hemipterans (suborder Auchenorrhyncha), that were the dominant arthropod on plots in early to mid-February (48% of total biomass) but largely absent by late March (6% of total biomass). We also only observed Lepidopterans in the early season and Orthopterans in the late season, but in both cases only a few individuals were detected. All of the other arthropod groups showed relatively consistent abundance between the early and late season. We found no significant differences in the change in total arthropod biomass on irrigated versus control plots (paired t-test, t = −0.16, p = 0.88, df = 9). We did not run separate statistical tests for each of the individual groups but the only group that showed a tendency to higher abundance post-irrigation on the experimental plots was Hymenoptera (Table S1).

### Avian Responses

Irrigation and control samples of redstarts each included five after second year (ASY) females, three second-year females (SY) and two SY males. Although 10 redstarts were measured prior to irrigation on treatment plots, we were only able to re-measure the condition post-irrigation on 4 individuals because 4 territories had turnover and the original occupant disappeared (see below for further detail on turnover) and in 2 cases we were unable to re-catch the territorial bird. We observed no instances of turnover on the control territories, and were able to recapture 8 of the 10 birds that were originally measured. Because of this difference, we did not conduct a paired statistical comparison of change pre- versus post-irrigation. A comparison of the mean change and error for the birds for which we had pre and post measures showed little evidence that individuals on irrigated plots were in better condition (mean residual mass and SE - irrigation: −0.08±0.103, n = 4; control: 0.04±0.153, n = 8). A two-sample t-test on all birds post-irrigation, including the new territory owners, showed no significant difference in body condition between irrigated (−0.081±0.101) and control plots (0.065±0.079, t = 1.15, p = 0.27, df = 16).

Four of 10 irrigation territories had a turnover of individuals, whereas no control territories did, a difference that was marginally significant (Fisher Exact Test, p = 0.087). On two of the territories, an ASY female and an SY male were each replaced with an ASY male. On the other two territories, we documented a continual turnover, with at least 3 different individuals showing territorial behavior on the plot between the end of irrigation and the departure for spring migration. Thus, while we found no evidence of an improved body condition on the irrigated plots, birds appeared to respond behaviorally to a perceived increase in the quality of experimental territories.

## Discussion

### Effects of Irrigation on Plant Productivity

Irrigation increased soil moisture levels on the experimental plots, which were approximately maintained at saturation, whereas soil moisture continued to decline as the dry season progressed on control plots. This difference resulted in significant variation among treatments in the extent of leaf abscission and the timing of new leaf growth. Within days after the onset of irrigation, the extent of leaf abscission decreased and the cumulative total was about 50% lower on experimental plots by the end of the dry season. Moreover, because our experiment began after about 30% of total control plot leaf abscission occurred, the true difference between treatments would almost certainly have been higher if the experiment had started earlier. Our project focused on the overall community response from a Neotropical migrant perspective and did not measure specific contributions from each species. However, the extent of leaf abscission was dominated by *H. campechianum,* by far the most common tree species in coastal scrub habitat in the study area. More detailed future studies that looked at the timing and extent for all tree species in scrub forest would be useful (e.g. [23], [24], [26]).

In addition to limiting leaf abscission, irrigation triggered leaf flushing in *H. campechianum*. Our third sampling period after the onset of the wet season in early May showed rapid and extensive leaf flushing with all sampled individuals on control plots exhibiting new growth. The artificially induced leaf flushing in February/March revealed a phenological shift but was also more limited and only 58% of sampled *H. campechianum* individuals had new growth. Overall, our results suggest that water status is a limiting factor for this tree species and a 200 mm dry season pulse of rainfall can trigger a response, although not equivalent to what is observed following the onset of wet season rains.

To our knowledge, only three other irrigation studies have been conducted in nearby regions of Central America. On Barro Colorado Island, Panama, Wright and Conejo [24] maintained soil water potentials at capacity through the dry season but only reported delayed leaf fall in a small proportion of the tree species examined, suggesting that plant water status was not a major factor affecting leaf phenology in most cases. In Western Mexico, Hayden et al. [31] used irrigation of varying intensities (0–200 mm) during the peak of the dry season and found positive relationships between irrigation intensity and leaf flushing for all tree species examined. Using even higher irrigation intensities (200–280 mm), Borchert [45] found a similar positive effect in dry forests of Guanacaste, Costa Rica, where irrigation was followed by the development of vegetative buds within 5 to 7 days and fully expanded leaves about two weeks thereafter. Similar to our findings for *H. campechianum,* Borchert’s [45] results also showed that the response to dry season irrigation was reduced relative to the rapid and synchronous response observed at the start of the wet season.

### Effects of Irrigation on Insect Abundance and Redstart Condition

We found little evidence that the plant response to irrigation translated to higher arthropod biomass or to improved body condition of American redstarts. Mean arthropod biomass was about 1.5 times higher on irrigated plots, but varied considerably among replicates and strongly declined in both treatments from mid-February to the end of March. At Barro Colorado Island, Panama, Wolda and Wright [36] conducted the only other irrigation experiment on Neotropical arthropods that we are aware of, and similarly found few effects of irrigation on arthropod biomass or diversity. Our results appear to be in contrast with non-experimental studies at our Jamaica site, where arthropod biomass and body condition of American redstarts and ovenbirds (*Seiurus aurocapilla*) were related to annual precipitation [15], [35]. N-15 values were higher for all groups on the irrigated plots, which suggests that arthropods and redstarts foraged at these sites, but we suspect that the lack of a significant trophic effect in our study was due to the small spatial scale of irrigation effects relative to the size of redstart territories and to a scale relevant to local arthropod populations. Approximately 1.6 million liters of fresh water were available in mid-February, but this level continued to drop through the dry season due to evaporation, which allowed us to use only 0.6 million liters throughout the experiment. In order to simulate 200 mm of dry season rainfall (the difference between a ‘wet’ year and a ‘dry’ year based on long-term data), this water limitation restricted us to irrigating 24 m diameter plots (∼450 m^2^), or about 20–30% of a typical redstart territory area.

Because most earlier studies were correlative it is not possible to conclude that precipitation and plant productivity are the limiting factors determining arthropod biomass during the dry season at these sites, although this hypothesis remains likely (e.g. [32], [33]). A minimal response to a sudden increase in plant productivity during the dry season may also reflect a limitation on the ability of arthropods to quickly adapt to this change. Alternatively, it is possible that arthropods responded to increased plant productivity but with a longer lag period such that we were unable to detect it by the time our sweep net surveys were conducted.

We observed several instances of redstart territory switching on the experimental sites, but none on control sites. In two cases, dominant, ASY males pre-empted territories from an adult female and a second year male, a result consistent with natural patterns observed on our Jamaica plots [7], [9], [46]. Two irrigated territory sites had no single territory holder. Instead, these sites experienced a continual turnover of individuals, with no single redstart holding a site for more than two weeks. Studies on other bird species, particularly hummingbirds, have shown the ability of individuals to adjust territory size, occupancy, or level of defense in relation to resource quantity or quality [47], [48]. Our findings suggest a similar pattern for redstarts in response to perceived territory quality as levels of food abundance became depleted towards the end of the dry season. Future irrigation studies should simultaneously collect data on foraging behavior and diet to examine the degree to which they correspond to both the timing and extent of plant productivity and variation in arthropod abundance.

In summary, our experimental irrigation study confirmed that water availability during the tropical dry season is a primary driver of plant productivity in lowland scrub forests of Jamaica. We were unable to document the effect of this productivity increase through higher trophic levels, probably due to the small spatial scale of our irrigation area. A winter redstart territory can be up to about 2500 m^2^ [41] and to irrigate 10 complete redstart territories with a pulse equal to 200 mm of rainfall, we would have needed a freshwater source of about 3 million liters. Larger scale irrigation experiments that encompass more of the individual species’ territory would be valuable, as would those that examine variation in phenology of different canopy and shrub species.

## Supporting Information

Table S1 **Canopy arthropod abundance by group on control and irrigation plots.** Early season refers to the period in late January/early February before irrigation, while late season refers to the period in late March/early April after irrigation had ended. Only the more commonly represented groups are shown here. Values are the sample mean mass (g) plus standard error and are based on measurements from 10 control and 10 experimental plots.
(DOCX)
