# Supplementary material for: The Effects of Experimental Irrigation on Plant Productivity, Insect Abundance and the Non-Breeding Season Performance of a Migratory Songbird
Source: PLoS One. 2013 Jan 25;8(1):e55114. doi: 10.1371/journal.pone.0055114 (PMC3555873; doi:10.1371/journal.pone.0055114)
Supplement: Table S1 — Canopy arthropod abundance by group on control and irrigation plots. Early season refers to the period in late January/early February before irrigation, while late season refers to the period in late March/early April after irrigation had ended. Only the more commonly represented groups are shown here. Values are the sample mean mass (g) plus standard error and are based on measurements from 10 control and 10 experimental plots. (DOCX) [file pone.0055114.s001.docx]

| Group | Early Season Control | Late Season Control | Early Season Irrigation | Late Season Irrigation |
| --- | --- | --- | --- | --- |
| Aranea | 3.78 + 0.79 | 2.19 + 0.55 | 3.63 + 1.46 | 2.59 + 1.29 |
| Hemiptera | 27.26 + 4.08 | 3.23 + 0.73 | 43.32 + 11.90 | 3.30 + 0.78 |
| Diptera | 0.59 + 0.31 | 0.16 + 0.08 | 0.74 + 0.30 | 0.44 + 0.18 |
| Hymenoptera (non-Formicidae) | 0.35 + 0.21 | 0.08 + 0.04 | 0.08 + 0.03 | 0.41 + 0.11 |
| Hymenoptera (Formicidae) | 0.15 + 0.14 | 0.33 + 0.18 | 0.67 + 0.54 | 2.01 + 1.41 |
| Lepidoptera | 10.97 + 10.97 | 0 | 4.77 + 4.07 | 0 |
| Coleoptera | 2.49 + 1.20 | 2.09 + 0.89 | 1.73 + 0.80 | 3.31 + 1.04 |
| Dictyoptera | 0.99 + 0.79 | 0.03 + 0.02 | 0.22 + 0.20 | 0.31 + 0.13 |
| Orthoptera^2^ | 0.36 + 0.26 | 15.46 + 12.30 | 0.09 + 0.06 | 9.63 + 8.27 |
| Neuroptera | 0.96 + 0.56 | 0.18 + 0.18 | 0.67 + 0.37 | 0 |
